# Patients with Chromosome 11q Deletions Are Characterized by Inborn Errors of Immunity Involving both B and T Lymphocytes

**DOI:** 10.1007/s10875-022-01303-8

**Published:** 2022-06-28

**Authors:** Elise J. Huisman, A. Rick Brooimans, Samone Mayer, Marieke Joosten, Louis de Bont, Mariëlle Dekker, Elisabeth L. M. Rammeloo, Frans J. Smiers, P. Martin van Hagen, C. Michel Zwaan, Masja de Haas, Marjon H. Cnossen, Virgil A. S. H. Dalm

**Affiliations:** 1 Department of Pediatric Hematology, Erasmus Medical Center Sophia Children’s Hospital, University Medical Centre Rotterdam, Rotterdam, the Netherlands; 2 Unit of Transfusion Medicine, Sanquin Blood Supply, Amsterdam, the Netherlands; 3 Laboratory Medical Immunological, Department of Immunology, Erasmus Medical Center, University Medical Centre Rotterdam, Rotterdam, the Netherlands; 4 Department of Clinical Genetics, Erasmus University Medical Center, Rotterdam, the Netherlands; 5 Department of Pediatric Immunology, University Medical Center Utrecht, Utrecht, the Netherlands; 6 Department of Pediatrics, Albert Schweitzer Hospital, Dordrecht, the Netherlands; 7 Department of Pediatrics, Bernhoven Hospital, Uden, the Netherlands; 8 Department of Pediatric Hematology, Leiden University Medical Center, Leiden, the Netherlands; 9 Department of Internal Medicine, Division of Allergy & Clinical Immunology, Erasmus University Medical Center, Rotterdam, the Netherlands; 10 Department of Pediatric Oncology, Erasmus Medical Center Sophia Children’s Hospital, University Medical Centre Rotterdam, Rotterdam, the Netherlands; 11 Department of Pediatric Oncology, Princess Máxima Center, Utrecht, the Netherlands; 12 Laboratory of Immunohematology Diagnostics, Sanquin Diagnostic Services, Amsterdam, the Netherlands; 13 Department of Hematology, Leiden University Medical Center, Leiden, the Netherlands; 14 Department of Clinical Transfusion Research, Sanquin Research, Amsterdam, the Netherlands

**Keywords:** Chromosome 11q, Jacobsen syndrome, Primary immunodeficiency, Inborn errors of immunity, B lymphocyte function, T lymphocyte function, Granulocyte function, Hypogammaglobulinemia, Children

## Abstract

Disorders of the long arm of chromosome 11 (11q) are rare and involve various chromosomal regions. Patients with 11q disorders, including Jacobsen syndrome, often present with a susceptibility for bacterial and prolonged viral and fungal infections partially explained by hypogammaglobulinemia. Additional T lymphocyte or granular neutrophil dysfunction may also be present. In order to evaluate infectious burden and immunological function in patients with 11q disorders, we studied a cohort of 14 patients with 11q deletions and duplications. Clinically, 12 patients exhibited prolonged and repetitive respiratory tract infections, frequently requiring (prophylactic) antibiotic treatment (*n* = 7), ear-tube placement (*n* = 9), or use of inhalers (*n* = 5). Complicated varicella infections (*n* = 5), chronic eczema (*n* = 6), warts (*n* = 2), and chronic fungal infections (*n* = 4) were reported. Six patients were on immunoglobulin replacement therapy. We observed a high prevalence of low B lymphocyte counts (*n* = 8), decreased T lymphocyte counts (*n* = 5) and abnormal T lymphocyte function (*n* = 12). Granulocyte function was abnormal in 29% without a clinical phenotype. Immunodeficiency was found in patients with terminal and interstitial 11q deletions and in one patient with terminal 11q duplication. Genetically, *FLI1* and *ETS1* are seen as causative for the immunodeficiency, but these genes were deleted nor duplicated in 4 of our 14 patients. Alternative candidate genes on 11q may have a role in immune dysregulation. In conclusion, we present evidence that inborn errors of immunity are present in patients with 11q disorders leading to clinically relevant infections. Therefore, broad immunological screening and necessary treatment is of importance in this patient group.

**Supplementary Information:**

The online version contains supplementary material available at 10.1007/s10875-022-01303-8.

## Introduction

Patients with 11q disorders are characterized by a partial deletion or partial duplication of the long arm of chromosome 11. Chromosomal alterations occur in various regions of 11q with deletion sizes ranging from < 5 to 20 Mb [1] and can occur with alterations in other chromosomes [2, 3]. Breakpoints arising within or distal to subband 11q23.3 and extending to the telomere give rise to Jacobsen syndrome (JS), alternatively referred to as 11q terminal deletion disorder [4, 5]. The estimated incidence is 1:100.000 births with a male:female ratio of 1:2 [1]. Clinical phenotype depends on the length, position, and type of the chromosomal alteration and may include cognitive impairment, cardiac malformation, increased bleeding tendency, and increased susceptibility for infections [1]. The latter is attributed to a humoral immunodeficiency with an abnormal B lymphocyte development and low memory B lymphocytes, resulting in hypogammaglobulinemia and an impaired response to immunization [6–9]. Although immunoglobulin replacement therapy (IgRT) significantly decreases the infectious burden [6], complications still occur [10]. Some authors suggest that low T lymphocyte counts [6–8, 11] or T lymphocyte dysfunction [7, 8, 11] may be causative. We hypothesized that granular dysfunction of neutrophils may also play a role, because patients with JS are also known to suffer from increased bleeding tendency due to delta storage pool defects [12], or giant alpha granules in platelets leading to the Paris-Trousseau syndrome [13]. The combination of platelet and neutrophil dysfunction is seen in other syndromes, such as Hermansky-Pudlak type 2 and grey platelet [14, 15].

Genetically, focus is on the transcription factors ETS1 and FLI1, both located on 11q24.3. For *ETS1*, experimental evidence in mice with a homozygous deletion shows that proliferation of B and T lymphocytes is decreased [16], and spontaneous apoptosis of T lymphocytes is increased [17–19]. Somatic loss of 11q23.3 has been found in B lymphocyte lymphoma (*MYC* negative), also indicating an effect on lymphocyte proliferation [20]. In humans, the role of *ETS1* was demonstrated in a recent case-report of a patient with a germline pathogenic frameshift mutation in *ETS1* in whom a decreased number of total and naïve B lymphocytes was found, as well as reduced presence of memory B lymphocytes. T lymphocyte number however was normal [21].

*FLI1* is a member of the E26 transformation-specific (*ETS*) gene family that shares a DNA-binding domain called *ETS* domain, which is responsible for sequence-specific DNA recognition of target promotors. This domain has a role in development of B and T lymphocytes [22]. Experimental evidence comes from *FLI1*-knockout mice, constructed with deletion of N-terminal region of *FLI1* (FLI1ΔNT). FLI1ΔNT mice exhibited thymic hypocellularity [23]. This defect was not associated with a specific subpopulation of thymocytes or apoptosis, implicating the role of *FLI1* in prethymic T lymphocyte progenitors [23]. The role of *FLI1* in B lymphocyte development has also been demonstrated by *FLI1*-knockout mice lacking the CTA domain (FLI1ΔCTA) [22, 24]. FLI1ΔCTA-homozygous mice show less splenic follicular B lymphocytes, and more transitional and marginal zone B lymphocytes [22]. Despite this fundamental evidence, clinically a variable phenotype-genotype is seen. In two families with an identical microdeletion in 11q24.2-11q24.3, one family demonstrated persistent lymphopenia and low IgG, but the other did not [25].

In order to evaluate immunological dysfunction in patients with 11q disorders, we prospectively investigated 14 patients focusing on clinical features, and tested both T lymphocyte and granulocyte function. We correlate our results with monosomy or trisomy of *ETS1* and *FLI1*.

## Patients and Methods

### Patient Population and Study Design

Patients were invited for this observational study by their treating physician or via the newsletter of the Dutch Chromosome 11 Network that represents patients with 11q deletions and 11q duplications. Institutional Review Board approval was obtained (MEC-2013–026) and the study was performed according to the Declaration of Helsinki. All patients were included after obtaining written informed consent according to local law and regulation.

Clinical data included number and type of infections, antibiotic treatment, and IgRT. Data was retrospectively derived from electronic patient files (EPF) after informed consent was given. Laboratory results for children were compared with age-dependent reference values for blood count [26], lymphocyte subsets [27], and IgGAM values (https://www.nvkc.nl/zoek-een-test/?id=237, https://www.nvkc.nl/zoek-een-test/?id=237). Results were recorded as normal when test results were in p5-p95 interval of the normal references. Molecular diagnosis was extracted from the EPF if the diagnosis was made by karyotyping or array. Patients were followed up by their own referring physician.

For laboratory tests a minimum of 0.5 mL of EDTA-blood, 0.5 mL serum and 6 mL heparinized blood were drawn by venepuncture. Cell count and indices were analyzed on Sysmex XN-9100 (Sysmex®). Total IgG, IgA, and IgM were investigated on Cobas 8000 Modular Analyzer Series (Roche Diagnostics®, Basel). Heparinized whole blood was used for flowcytometric measurement of lymphocyte count (FACSCanto II, BD®, USA).

### *Detection of Antigen-Specific CD4*.^+^*T Lymphocytes*

Antigen-specific CD4 + T lymphocytes were measured using a commercially available kit (Act-T4 Cell™, Cytognos, Spain) that is based on the “OX40-assay.” [28] Heparinized whole blood was diluted 1:1 with RPMI-1640 (Gibco, Paisley, UK) culture medium supplemented with 10% fetal calf serum (Gibco), L-glutamine 2 mM (BioWhittaker), and penicillin and streptomycin (100 IU/mL; BioWhittaker), then aliquoted into 500μL volumes in sterile capped 5-mL polystyrene flow cytometry tubes. The following conditions were used for each assay: no exogenous stimulation (negative control), PHA (2.5 μg/mL), Staphylococcal enterotoxin B (SEB; Sigma-Aldrich) (0.5 μg/mL), and activation with tuberculin PPD (4 μg/mL), tetanus toxoid (1 μg/mL), diphtheria toxoid (10 μg/mL), Candida lysate (4 μg/mL), CMV lysate (2 μg/mL), HSV1 lysate (1 μg/mL), HSV2 lysate (1 μg/mL), and VZV lysate(1 μg/mL). After 44-–48-h stimulation in a humidified atmosphere of 5% CO2, samples were vortexed and 100μL of each culture was stained with 20 μL of the 4-antibody mixture provided in the Act-T4 Cell™ kit (CD3-PerCP-Cy5.5, CD4-FITC, CD25-APC, and CD134-PE, Cytognos) and incubated for 15 min at room temperature. The assay was protected from light. After incubation, 1 mL of erythrocyte lyse-non-wash solution provided in the Act-T4 Cell™ kit was added to each tube, mixed, and incubated for 10 min at room temperature, still protected from light. After lysis 10,000 CD3-positive events were acquired on flow cytometer (FACSCanto II) and analyzed with Infinicyt software (Cytognos). Quantification of CD3 + CD4 + CD25 + CD134 + T lymphocytes were calculated by setting gate coordinates on the negative control sample to equal 0, 1% CD3 + CD4 + CD25 + CD134 + events as a percentage of all CD3 + CD4 + events. These gate coordinates were applied to subsequent antigen tubes to produce a percentage value of CD25 + CD134 + double positive events. Normal reference values were determined in a cohort of 40 healthy controls.

### Determination of Granulocyte Phagocytic Activity–Phagotest

The granulocyte phagocytic activity was measured in whole heparinized blood using a commercially available kit (Phagotest®, Glycotope Biotechnology, Germany) according to manufacturer’s instructions. In short, FITC-labeled opsonized *Escherichia coli* bacteria were added to whole blood and incubated for 10 min at 37 °C (experimental tube) or 0 °C (negative control tube). After incubation, the reaction was stopped, erythrocytes were lysed, and the DNA staining solution was added. Fluorescence of samples was measured by flow cytometer (FACSCanto II) in < 60 min after the last reagent had been added. Data were acquired by FACSDiva software (BD Biosciences) and analyzed by Infinicyte software. The Phagotest is performed with the involvement of fluorescein-stained *E. coli* bacteria which are phagocytized by the cells. The test determines the percentage of granulocytes and their phagocytic activity, i.e., the number of bacteria absorbed by a single cell in terms of mean fluorescence intensity (MFI).

### Determination of Oxidative Burst Activity of Granulocytes–Phagoburst

A respiratory burst assay was performed using a commercially available kit (Phagoburst®, Glycotope Biotechnology, Germany) according to manufacturer’s instructions. Opsonized *E. coli* bacteria (experimental tube), or phorbol 12‐myristate 13‐acetate (PMA, positive control tube) or washing solution (negative control tube) were added to whole blood and incubated for 10 min at 37 °C. Following incubation, dihydrorhodamine (DHR 123) was added for 10 min, erythrocytes were lysed, and DNA-staining solution was added. Dihydrorhodamine 123 becomes fluorescent when oxidized by reactive oxygen species, and its fluorescence was measured in less than 30 min after the last reagent had been added by flow cytometry. The test determines the percentage of active cells, and the respiratory burst intensity within a single cell in terms of MFI.

### Statistical Analysis

We used descriptive statistics to summarize baseline characteristics of the study population. In case of a skewed distribution, data are presented as median and interquartile range (IQR). In case of a normal distribution, data are presented as mean and standard deviation (SD) or range. Categorical data are presented as numbers with percentages and range of minimum and maximum value.

Reference values of healthy controls were available for the various antigens that were used as stimuli in the OX40 test. The results of the T lymphocyte function tests were considered abnormal when the results were < p5 of healthy controls. Also for both granulocyte function tests obtained, reference values of healthy controls were used. The phagocytic and oxidative burst capacity results were considered abnormal when the results were < p5 of healthy controls. Statistical data analyses were performed using SPSS version 21.0 (IBM, Armonk, NY, USA).

## Results

### Patient Characteristics

Sixteen patients were identified, one patient declined participation, and one passed away before study initiation. We included 14 patients (*P*), with a median age of 9.1 years (IQR 5.6–17.4; range 0–40), and four males (29%). Patients 1, 2, 7, and 11 of our cohort have been investigated in an earlier publication by Dalm et al. They correspond with the patients 2, 6, 5, and 4 [6]. Twelve presented with an interstitial or terminal 11q deletion, two with 11q duplication, one interstitial, and one terminal. The extent and location of the involved regions varied widely, and in three patients’ additional chromosomal abnormalities were observed. During follow-up, one patient (P7) passed away (Table 1).

**Table 1:**
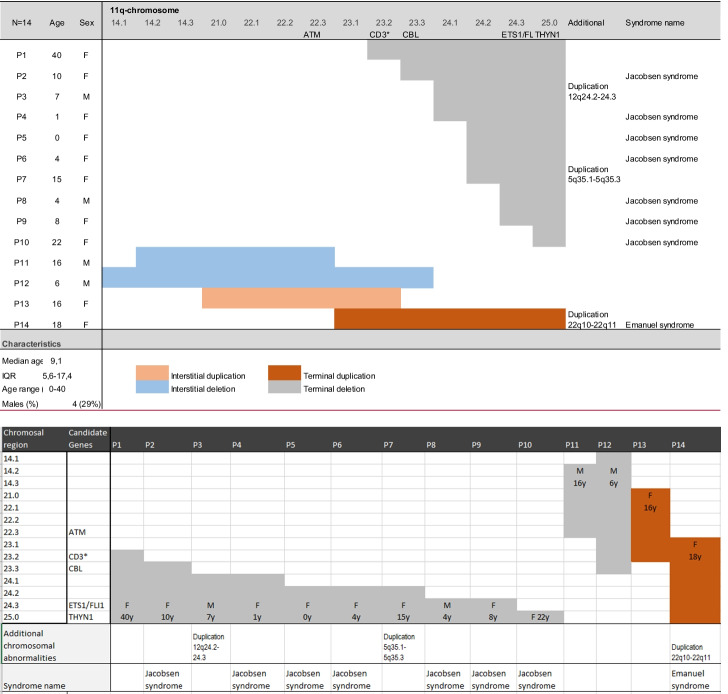
Patient characteristics of 14 patients with 11q disorders

Clinically*,* all patients were immunized according to recommendations of the Dutch National Institute for Public Health and the Environment (RIVM) (https://rijksvaccinatieprogramma.nl/vaccinaties/vaccinatieschema). This does not involve varicella or rotavirus vaccines. Frequent infections were reported in the majority of patients, especially during childhood (12/14; 86%) and most often affecting upper and lower respiratory tract (RTI). These infections required frequent or prophylactic antibiotic treatment (7/12; 70%). Nine required ear-nose-throat (ENT) interventions such as ear tubes or adeno-tonsillectomy (9/14; 64%). Five required (5/14; 36%) therapy with inhaled β2 agonists, and three (3/14; 21%) were hospitalized for oxygen supplementation as supportive measures in case of infectious complications. Six patients (6/14; 43%) were on intravenous or subcutaneous IgRT because of hypogammaglobulinemia. All six patients on IgRT reported less infections and an improved physical condition (Table 2).

**Table 2:** Immunological symptoms and comorbidities of 14 patients with 11q disorders

P	Age	IgRT	Viral infections	Bacterial infections	Eczema	Warts	Mycosis	Comorbidities
On immunoglobulin replacement therapy (IgRT)								
1	40	Yes	Frequent prolonged viral upper respiratory infections (URTI); frequent ear-nose-throat infections (ENTI) requiring tubes; varicella-zoster virus infection (VZV): caregiver does not recall	Frequent oral antibiotics; paronychia requiring drainage	None	Multiple on hands and feet, needing. Gardasil	None	Increased bleeding tendency; asymmetric aortic valve; eyelid surgery; Hearing aids; lymphangiodysplasia of lower legs; moderate developmental delay
2	10	Since age of 6	Frequent URTI; frequent ENTI requiring tubes; frequent rehydration after URTI and gastro-intestinal (GI) infections; Severe VZV	Frequent oral antibiotics; paronychia requiring drainage; bacterial superinfection of the skin during VZV requiring local and iv AB	None	None	Fungal nails and vaginal mycosis, chronic use of anti-mucosal treatment	Increased bleeding tendency; craniosynostosis repairment; eyelid surgery; Type 1 diabetes mellitus (11y); moderate-severe developmental delay
3	7	Yes	Frequent URTI and ENTI; viral bronchitis and BHR requiring hospitalization for salbutamol and oxygen; mild-moderate VZV	Frequent oral antibiotics and prophylaxis until age of 6y	None	None	None	Increased bleeding tendency; large VSD; Eyelid surgery; Nissen surgery for reflux disease (GERD); moderate-severe developmental delay
7	15	Since age of 10y	Frequent URTI; frequent ENTI requiring tubes; Severe H1N1 influenza in 2009, needing Tamiflu; recurrent hospitalization for dehydration due to GI-infections; moderate VZV	Frequent AB; Hospitalization for sepsis* (occurred under IgRT)	None	None	None	Increased bleeding tendency; large VSD; Craniosynostosis repairment; eye lid surgery; severe GERD; celiac disease; severe developmental delay. epilepsy; sudden death (16y)
11	16	Since age of 5y	Frequent URTI and ENTI; severe RSV and parainfluenza infection requiring oxygen. BHR, needing salbutamol and ICS; hospitalization for dehydration due to rotavirus; severe VZV	Frequent AB and prophylaxis; once iv for LRTI. Paronychia requiring topical AB. Balanitis, requiring circumcision; bacterial superinfection of the skin during VZV	Chronic, resolved under IgRT	None	Pityriasus rosacea, fungal nails needing oral terbinafin	Increased bleeding tendency; left pulmonary artery stenosis; mild developmental delay
14	18	Since age of 14y	Frequent URTI; frequent ENTI requiring tubes; frequent oral aphthous stomatitis; severe VZV	Frequent AB; bacterial superinfection of the skin during VZV requiring AB	Chronic, resolved under IgRT	One persistent on toe	Topical ketoconazole for frequent diaper dermatitis	Increased bleeding tendency; congenital hip dysplasia; moderate-severe developmental delay
Not on immunoglobulin replacement therapy								
4	1	No	No prolonged or complicated viral infections	No infections	None	None	None	Increased bleeding tendency; multiple VSDs; Feeding difficulties; mild developmental delay
5	0	No	No prolonged or complicated viral infections	No infections	None	None	None	Increased bleeding tendency; craniosynostosis repairment; normal development
6	4	No	Frequent URTI; dehydration due to GI-infections; Severe VZV	Bacterial superinfection of the skin during VZV	Chronic in hair, axillary regions and umbilical area	None	Skin fungal dermatitis, needing miconazole	Increased bleeding tendency; strabismus; benign hydrocephalus; mild speech delay
8	4	No	Frequent URTI; frequent ENTI requiring tubes; moderate-severe VZV	Frequent AB and prophylaxis	Moderate	None	Chronically affected, between toes, needing miconazole	Increased bleeding tendency; normal development
9	8	No	Frequent URTI frequent ENTI requiring tubes; BHR, requiring salbutamol; once hospitalized for oxygen replacement during viral LRTI. Severe VZV	Frequent AB; bacterial superinfection of the skin during VZV requiring AB	Chronic	Chronically affected toe-nails	None	Normal bleeding tendency; variant HLHS; ADHD; mild developmental delay
10	22	No	Frequent URTI and ENTI; BHR requiring salbutamol; once hospitalization for LRTI Moderate VZV	Frequent AB	Frequent seborrheic eczema around ears and in hair	None	None	Increased bleeding tendency; ASD, VSD, MS, ODB, and pulmonary hypertension Hearing aids; fever-related convulsions Moderate developmental delay
12	6	No	Frequent URTI; Frequent ENTI requiring tubes; BHR requiring salbutamol; Severe pseudocroup requiring epinephrin; severe picornavirus and rhinovirus; no VZV	Frequent AB and AB prophylaxis	Yes	None	None	Increased bleeding tendency; vision loss after vitreous hemorrhage; Severe MI Surgery for cleft palate; Feeding difficulties; retrognathia; horse shoe kidney; osteoporosis; neonatal apneas and hypotonia. Epilepsy; moderate-severe developmental delay
13	16	No	Frequent URTI; frequent ENTI requiring tubes; no VZV	Sporadic antibiotics; frequent urinary tract-infections, once complicated by pyelonephritis			Frequent diaper dermatitis, needing miconazole	No bleeding tendency Fever-related convulsions Moderate-severe developmental delay

*Abbreviations: AB *antibiotics, *ADHD *attention deficit hyperactivity disorder, *ASD* atrial septum defect, *BHR* bronchial hyperreactivity, *ENT* ear-nose-throat, *GERD* gastro-esophageal reflux disease, *GI* gastro-intestinal, *HLHS* hypoplastic left heart syndrome, *ICS* inhalation corticosteroids, *IgRT* immunoglobulin replacement therapy, *iv* intravenous, *LRTI* lower respiratory tract infections, *MI* mitral valve insufficiency, *MS* mitral valve stenosis, *ODB* open duct of Botalli, *RSV* respiratory syncytial virus, *URTI* upper respiratory tract infections, *VSD* ventricular septum defect, *VZV* varicella-zoster virus, *y* year

Complicated viral infections leading to hospitalization were confirmed for respiratory syncytial virus (RSV), picornavirus, para-influenza virus, and rhinovirus. Complicated and prolonged varicella zoster virus (VZV) infection occurred in 5/14 (36%). Four of 14 patients (29%) suffered from gastro-enteritis requiring hospitalization because of dehydration. Rotavirus was confirmed in two of these episodes. Fungal infections, most often in the diaper area or the nails, were reported in 4/14 (29%), persistent warts by two patients (2/14; 14%), and chronic eczema by six (43%), which resolved in patient P11 and 14 after IgRT was initiated.

Immunological laboratory results are presented in Table 3. Low leucocyte counts were seen in two patients (P2, 7): 2.8 and 3.2 × 10^E^9/L respectively (normal range 3.8–9.8 × E10^9^/L). In five (36%), an absolute lymphopenia was observed (mean 0.78; absolute range 0.63–0.95 compared to age-dependent normal ranges).

**Table 3:** Immunological analysis of 14 patients with 11q disorders

Patient	Leuco (10^E^9/L)	Neutro (10^E^9/L)	Lympho (10^E^9/L/%)	IgA (g/L)	IgG* (g/L)	IgM (g/L)	Tc total, CD4 + , CD8 + (10^E^9/L)			CD4/CD8 ratio	Bc total (10^E^9/L)	NKc total (10^E^9/L)	Granulocyte function		
													Chemotaxis	Phagocytosis	Oxidative burst
On immunoglobulin replacement therapy															
1	8.7	7.71	**0**.**68 (8%)**	1.93	9.1*	**0**.**22**	**0**.**58**	0.49	**0**.**09**	High	**0**.**04**	**0**.**09**	Normal	Normal	Normal
2	**2**.**8**	2.02	**0**.**63** (23%)	0.82	12.2*	** < 0**.**05**	**0**.**55**	0.32	**0**.**23**	Normal	**0**.**04**	**0**.**03**	Normal	Normal	**Inadequate**
3	6.6	3.3	2.90 (44%)	**0**.**40**	**3**.92*	**0**.**13**	**0**.**11**	**0**.**07**	**0**.**04**	Normal	**0**.**14**	**0**.**08**	-	-	-
7	**3**.**2**	1.94	**0**.**95** (30%)	0.91	17.9*	**0**.**24**	0.85	0.44	0.30	Normal	**0**.**09**	**0**.**13**	Normal	Normal	**Inadequate**
11	6.7	4.25	1.63 (24%)	**0**.**47**	**0**.8*^^^	**0**.**21**	1.14	0.74	0.38	Normal	0.21	0.40	Normal	Normal	Normal
14 T†	17.5	13.2	2.08 **(12%)**	0.65	**5**.2*	0.97	1.53	1.06	0.47	Normal	**0**.**11**	0.46	Normal	Normal	Normal
Not on Immunoglobulin Replacement Therapy															
4	6.2	2.9	2.71 (44%)	0.31	6.8	**0**.**20**	1.99	1.28	0.65	Normal	0.83	**0**.**17**	-	-	-
5	6.2	1.76	3.62 (58%)	0.26	3.8	**0**.**14**	1.71	1.17	0.50	Normal	0.50	0.29	-	-	-
6	4.9	2.77	1.76 (36%)	1.40	11.3	0.30	1.27	0.62	0.60	**Low**	0.46	**0**.**10**	Normal	Normal	Normal
8	6.1	4.8	**0**.**90 (15%)**	0.96	7.7	0.44	**0**.**58**	**0**.**22**	0.32	**Low**	**0**.**15**	**0**.**14**	Normal	Normal	Normal
9	6.0	5.0	**0**.**76 (13%)**	1.59	8.1	0.45	**0**.**63**	**0**.**27**	0.35	**Low**	**0**.**17**	**0**.**06**	**Inadequate**	Normal	Normal
10	5.5	3.5	1.63 (29%)	2.07	10.5	0.55	1.23	0.69	0.48	Normal	**0**.**17**	0.19	Normal	Normal	Normal
12	13.3‡	8.1	3.84 (28%)	**0**.**41**	8.0	**0**.**30**	3.12	2.27^$^	0.79	Normal	0.64	0.26	Normal	Normal	Normal
13 T	5.8	2.8	2.16 (37%)	1.83	11.7	1.28	1.67	0.89	0.68	Normal	0.35	0.44	**Inadequate**	Normal	Normal
Mean	7.11	4.58	1.88	1.00	8.12^#^	0.42	1.21	0.64	0.42		0.28	0.20			
SD	3.88	3.17	1.07	0.64	2.69	0.34	0.77	0.38	0.22		0.24	0.14			

Bold values mean < p5 age-adapted reference values

*Abbreviations: Bc* B lymphocyte, *CD* cluster of differentiation, *Ig* immunoglobulin, *Leuco* leucocyte, *Lymph* lymphocyte, *Neutro* neutrophil granulocyte, *NKc* natural killer cell, *SD* standard deviation, *T* trisomy, *Tc* T lymphocyte

^*^IgG during immunoglobulin replacement therapy. If pre-substitution IgG was know, this is mentioned. Otherwise the IgG at inclusion time is mentioned

^^^At 3 months of age, IgG total was 0.8, this increased until 3.1 at age of 3 years. Patient started IgG substitution in 2004

^†^T = 11q trisomy

^‡^Patient had a viral bronchitis at time of inclusion, but retrospectively total leucocyte count was always higher than the age-adjusted reference values

^#^Mean calculated on > 3 values pre- or non-IgRT

The total numbers of T lymphocytes and B lymphocytes were normal or elevated in four patients (4/14; 29%), and all had IgG levels within the reference range. Total B lymphocyte counts were low in eight patients (57%, normal reference > 0.20 × 10E^9^/L). In five (5/8, 63%), T lymphocyte numbers were also low. Remarkably, all eight patients with low B lymphocyte counts presented with clinically relevant and increased number of infections, regardless of IgG levels (P1-3.7–10.14). The absence of an association between total number of B lymphocytes and function is illustrated in P11, who had a hypogammaglobulinemia from infancy with normal but steadily decreasing B lymphocyte count up to the age of 18 years (from 1.68 × 10E^9^/L at age 1, to 0.21 × 10E^9^/L at age 18). Low IgG levels were found in six patients (6/14; 43%), all corrected on IgRT. A concomitant low IgM (2/6) sometimes with combined low IgM and IgA (2/6) was seen in four (4/6, 67%). Only low IgM levels were seen in three patients (3/14; 21%), of whom two were < 2 years of age at time of inclusion. One patient had a low combined IgA and IgM (P12).

Total T lymphocyte counts were low in five patients (5/14, 36%). In one patient, both CD4 + and CD8 + T lymphocytes were decreased (1/5), while in two only CD4 + and in two only CD8 + T lymphocytes were decreased (normal reference CD4 +  > 0.3 × 10E^9^/L, CD8 +  > 0.2 × 10E^9^/L), resulting in an abnormal CD4:CD8 ratio in four (P1, 6, 8, 9). T lymphocyte function was evaluated in 13 patients using an OX40-based test. (Table 4) In general, only two patients showed a normal response to all stimuli (P6, 13). T lymphocytes from ten patients showed at least one abnormal response to the various stimuli (83%). For the viral agonists, T lymphocytes of all patients on IgRT failed in CMV response (5/5, 100%). Also, T lymphocytes of three patients (not on IgRT, but with a history of frequent viral upper airway and ENT infections) failed on CMV-response (P9-10, 12). As this assay is a functional test, diminished responses are either due to an intrinsic T lymphocyte defect, or due to the fact that a patient has not yet encountered CMV. Unfortunately, data of seroconversion were missing. Medical history was certain for 13 patients for VZV. Of these 13, three patients with a history of VZV infection demonstrated an abnormal T lymphocyte response (P3, 7, 2; 3/12; 25%). Despite the fact that all patients were vaccinated, an impaired response to the conjugated vaccine against tetanus was observed in two patients (P1&2; 2/12; 7%). All responded well to diphtheria and also to tuberculin. For the Toll-like receptor (TLR) agonists, five patients showed abnormal result after aspecific T cell stimulation with the superantigen SEB (through TLR2) or the mitogen PHA (through TLR4) (P3,1,7,2,9; 4/12, 33%).

**Table 4:** Tc function assay analysis of 13 patients with 11q disorders

Patient	CD4 + Tc^#^	Sample control	PHA	SEB	CMV	Candida	TT	Tuberculin	Diphtheria	HSV 1	HSV 2	VZV	Interpretation
On immunoglobulin replacement therapy													
1	0.49	0.20	**1**.**43**	**8**.**60**	**0**.**31**	0.18	0.19	0.19	0.25	**0**.**39**	0.32	**0**.**25**^**&**^	Inadequate response
2	0.32	0.09	12.95	**1**.**59**	**0**.**07**	0.18	0.20	0.15	0.12	**0**.**15**	0.11	***0***.***13***	Inadequate response
3	0.37	0.23	22.74	**3**.**83**	**0**.**14**	0.65	1.86	0.16	0.20	**0**.**16**	0.27	***0***.***39***	Inadequate response
7	0.44	0.13	**4**.**38**	**7**.**15**	**0**.**13**	0.63	0.97	0.24	0.64	**0**.**11**	**0**.**07**	***0***.***15***	Inadequate response
11	0.74	0.06	54.36	16.36	**0**.**06**	3.83	8.20	1.59	2.38	11.06	6.84	*3*.*55*	Inadequate response to CMV only
14 T	1.06	0.09	26.21	15.01	**0**.**12**	7.20	3.50	2.38	4.10	**0**.**20**	0.13	*2*.*07*	Inadequate response to CMV and HSV1 only
Not on immunoglobulin replacement therapy													
4	0.65	0.03	33.79	17.63	1.67	0.13	0.24	0.29	**0**.**02**	**0**.**09**	**0**.**07**	**0**.**15**	Inadequate response
6	0.58	0.22	13.14	17.36	1.44	0.64	3.10	0.45	2.41	**0**.**13**	0.39	*1*.*24*	Inadequate response HSV1 only
8	**0**.**22**	0.06	15.25	**9**.**43**	**0**.**69**	0.30	0.21	1.58	0.18	1.81	0.74	***0***.***60***	Inadequate response
9	**0**.**27**	0.07	**3**.**03**	16.2	1.39	0.09	0.27	0.14	0.64	**0**.**12**	0.26	***0***.***63***	Inadequate response
10	0.69	0.34	29.71	18.22	**0**.**16**	0.96	1.10	0.29	0.65	**0**.**35**	0.51	*1*.*29*	Inadequate response to only CMV and HSV1
12	2.27	0.07	10.13	18.74	**0**.**15**	0.82	3.04	0.69	2.20	**0**.**10**	0.15	**0**.**16**	Inadequate response
13 T	0.89	0.35	23.91	19.96	8.94	12.02	2.34	9.82	3.91	13.99	8.93	3.68	Normal response
	**P5**	0.02	8.69	11.77	1.35	0.08	0.13	0.06	0.04	1.70	0.10	0.99	
HC *n* = 40	**Mean**	0.08	27.60	20.67	6.69	1.05	2.81	1.45	0.68	6.96	2.50	2.22	
	**P95**	0.19	61.17	33.78	12.29	9.14	8.47	5.85	3.70	15.52	5.88	7.73	

Underscored italic numbers in VZV column demonstrated patients that have encountered VZV based on medical history

Bold < p5 reference values based on 40 healthy controls (HC)

*PHA* phytohemagglutinin, *SEB* staphylococcal enterotoxin B, *CMV* cytomegalovirus, *HC* healthy controls, *N* number, *T* trisomy, *TT* tetanus toxoid, *HSV* herpes simplex virus, *VZV* varicella-zoster virus

^#^CD4 + Tc number in sample taken for functional T lymphocyte test. This can slightly differ from Table 2 due to physiological variation in blood cell numbers

^&^Caregiver does not recall if patient has had VZV

Neutrophil granulocyte counts were normal in all 14 patients. Neutrophil granulocyte function was abnormal in four patients (P13, 7, 2, 9; 4/14, 29%). Two patients (P2, 7), who responded with a lower than normal oxidative burst, presented with a history of frequent infections and antibiotic usage, but none of the patients had opportunistic infections with microbes that are pathognomonic for neutrophil dysfunction, e.g., invasive *S. aureus*, *Aspergillus*, *Nocardia*, *Burkholderia*, or *Serratia*. Both patients are currently on IgRT. The two other patients (P9, 13) showed abnormal responses in chemotaxis, of whom patient 13 (P13) with a trisomy only reported chronic (diaper) dermatitis and ENT infections in infancy, and patient 9 (P9) reported an increased infectious burden in ENT and RT, but is not on IgRT due to normal IgG levels.

*ETS1* and *FLI1* involvement did not correlate to patients with immunodeficiency of B nor T lymphocyte dysfunction in our cohort (see Table 5).

**Table 5:** *ETS1* and *FLI1* involvement in 14 patients with 11q-disorders

Patient	Age	Infectious burden	Bc count	Tc count	CD4:CD8 ratio	IgG	Tc function	*FLI1*	*ETS1*
On immunoglobulin replacement therapy									
1	40	↑↑	↓	↓	Abnormal	↓	Inadequate	Deleted	Deleted
2	10	↑↑	↓	↓	Normal	↓	Inadequate	Deleted	Deleted
3	7	↑↑	↓	↓	Normal	↓	Inadequate	Deleted	Deleted
7	15	↑↑	↓	Normal	Normal	↓	Inadequate	Deleted	Deleted
11	16	↑↑	Normal	Normal	Normal	↓	Inadequate	Normal	Normal
14	18	↑↑	↓	Normal	Normal	↓	Inadequate	Duplicated	Duplicated
Not on immunoglobulin replacement therapy									
4	1	Normal	Normal	Normal	Normal	Normal	Inadequate	Deleted	Deleted
5	0	Normal	Normal	Normal	Normal	Normal	Inadequate	Deleted	Deleted
6	4	↑↑	Normal	Normal	Abnormal	Normal	Inadequate	Deleted	Deleted
8	4	↑↑	↓	↓	Abnormal	Normal	Inadequate	Deleted	Deleted
9	8	↑↑	↓	↓	Abnormal	Normal	Inadequate	Deleted	Deleted
10	22	↑↑	↓	Normal	Normal	Normal	Inadequate	Normal	Normal
12	6	↑↑	Normal	Normal	Normal	Normal	Inadequate	Normal	Normal
13	16	↑	Normal	Normal	Normal	Normal	Normal	Normal	Normal

*Bc* B lymphocyte, *CD* cluster of differentiation, *Ig* immunoglobulin, *P* patient number, *Tc* T lymphocyte

## Discussion

We present 14 patients with a variety of 11q disorders. We demonstrated an increased vulnerability to infections in all, but with varying clinical impact. Infections started in infancy with recurrent and/or chronic ENT and airway infections, leading to high consumption of not only antibiotics, but also of inhalation therapy, hospitalization, and the performance of ENT surgery. Vulnerability for both viral and bacterial infections is illustrated by the finding that almost half of patients suffered from VZV infections with multiple skin lesions leading to secondary bacterial skin infections. Also, other skin manifestations, including eczema, dermatitis, fungal skin and nail infections, and warts, were encountered regularly.

We confirm low B lymphocyte numbers in 57% (8/14), as well as B lymphocyte dysfunction resulting in hypogammaglobulinemia in 64% (9/14) of patients in infancy or at older age, necessitating IgRT in 6/9) [6, 7]. We underline the relevant finding that low IgG is not always accompanied by low total B lymphocyte counts and vice versa, as demonstrated in patient 11 (P11) [6, 11].

We demonstrate that T lymphocyte dysfunction occurs in the majority of patients with 11q disorders. We showed that decreased T lymphocyte counts or abnormal CD4/CD8 ratio occur in 43% of our cohort and abnormal in vitro T lymphocyte activation in 92% (12/13). Especially, the abnormal responses to stimuli such as PHA, SEB, diphtheria, CMV, and VZV are clinically relevant. Unfortunately, we were not able to test seroconversion for CMV. The abnormal response may be due to naivety toward CMV, HSV1, or VZV in four patients, still giving an abnormal test result in 8. Qualitative T lymphocyte defects have been reported in cases before [7, 11, 29], but none of the applied stimulation tests in these studies was identical to the assays we used, which makes comparisons between results difficult. We conclude that inborn errors of T lymphocyte immunity regularly occur, which was suggested in previous case series [6, 7, 9, 11, 30, 31].

Finally, although we demonstrate a disturbed granulocyte function in vitro, these findings may be interpreted as mild or irrelevant as none of the patients experienced typical infections with, e.g., *Aspergillus* or invasive *S. aureus*. It therefore remains unclear whether the need for antibiotics due to a bacterial superinfection after VZV (P2, 6, 9, 11, 14; 56%) is due to hypogammaglobulinemia only, or combined with a potential granular dysfunction (only P2, 11, 14 are on IgRT). In a single patient on IgRT, hospitalization for possible septicemia was documented. In this patient, an isolated abnormal response in the Phagoburst assay was found in granulocyte function analysis (P7). Our current results neither clearly support nor rule out the hypothesis that granulocyte dysfunction is responsible for the high incidence of infections, as more research in this area is indicated. Novel diagnostic tools as electro-microscopy (EM) may be promising [15].

*ETS1* and *FLI1* involvement did not correspond with abnormal B or T lymphocyte number or function. While monosomy was seen in 9 patients (P1-9) and trisomy in 1 (P14), an abnormal B or T lymphocyte number was only seen in 7. On the other hand, low T lymphocyte numbers (P10) and abnormal function was seen in 3 patients (P10-12) in which *ETS1* and *FLI1* copies were normal (Table 5). This variable phenotype-genotype may be due to incomplete penetrance, as is suggested in congenital microdeletions in two families with an identical microdeletion in 11q24.2-11q24.3. One family demonstrated persistent lymphopenia and low IgG, the other did not [25]. But, this variability may also be due to other genes or gene clusters that are located on 11q and are involved in cell proliferation and differentiation. Genes of interest are *ATM*, *CD3*, *CBL*, and *THYN1*.

*ATM* is located on 11q22.3 (OMIM #607,585). Homozygous or compound heterozygous mutations cause ataxia-telangiectasia (AT) (https://omim.org/entry/607585). AT is characterized by cerebellar ataxia, telangiectasia, and immune defects, such as reduced antibody levels, lymphopenia, and abnormalities in T lymphocyte maturation. Also, variant AT with residual ATM protein expression and kinas -activity causes a milder phenotype. In variant AT, only one truncating mutation is found, together with a compound missense, splice variant, or leaky mutation [32, 33].

The clusters of ***CD3EAP, CD3D, CD3E, CD3G, CD3Z*** are all located on 11q23.2, and regulate the synthesis of T lymphocyte antigen receptor chains: Tγ, Tδ, Tε, Tζ (https://omim.org). During development, the CD3 complex plays an important role in the transition of thymocytes from immature precursors to the final mature CD4 + or CD8 + T lymphocytes [34]. Pathogenic variants in any of these genes may cause blockage at the stage of CD4 + /CD8 + lymphocytes, resulting in reduced Tγδ lymphocytes [34].

***CBL***, located on 11q23.3, encodes for an E3 ubiquitin ligase acting as a regulator in the tyrosine kinase signaling pathway (https://omim.org/entry/165360). *CBL* plays a role in lymphocyte development and activation by regulating development of the thymocyte, and by regulating receptor signaling thresholds for B lymphocyte maturation [35, 36]. Besides single nucleotide variants, Hanson et al. describe this entity as the result of uniparental isodisomy of 11q23, underlining the possible influence of this gene for the clinical phenotype of 11q disorders [37].

***THYN1***, located on 11q25, encodes for thymocyte nuclear protein 1, which is expressed in the thymus (https://omim.org/entry/613739). This gene may be involved in the induction of apoptosis or T lymphocyte regulation [38, 39]. As yet, we are not aware of diseases in humans caused by mutations this gene.

## Study Limitations

Our study has several limitations. Firstly, we describe only a small cohort in whom the genetic defects vary considerably and concurrent chromosomal abnormalities occur. This reflects medical practice as 11q disorders are a rare contiguous gene syndrome, but extrapolation of results to other 11q disorders is challenging. In our cohort, seven patients had the “classical” Jacobsen syndrome (11q23.3 terminal deletion syndrome). Two patients had an interstitial 11q deletion. We have chosen to include these 2 patients as well, because both show the same vulnerability to infections and bleeding as terminal 11q patient exhibit. It is unknown if this is due to other candidate genes or due to downstream loss of function resembling terminal 11q deletion patients. There are hardly any data available on this rare entity, and with our results we like to raise awareness that inborn errors of immunity may be seen in patients with interstitial 11q disorders. The same accounts for the two 11q duplication patients. Three patients had additional chromosomal abnormalities, of whom P14 had a combination also referred to as Emanuel syndrome. No clear inborn errors of immunity have yet been reported to occur in Emanuel syndrome, but frequent ENT infections are reported in cases and are mentioned by the unique patient organization to occur “in a minority.” (https://www.rarechromo.org/media/information/Chromosome%2011/Emanuel%20syndrome%20FTNW.pdf) [40]. The trisomy of 11q23.3 in our patient may give rise to hypogammaglobulinemia and T lymphocyte dysfunction, but the 22q11 region can also cause T lymphocyte dysfunctions or T lymphocytopenia as is seen in other chromosome 22q abnormalities and in one case report of a child with a 22q11.2 microduplication [41]. No hypogammaglobulinemia was seen in this child [42, 43]. We therefore feel that it is unclear whether the hypogammaglobulinemia and abnormal T lymphocyte function in our patient are only due to terminal 11q duplication. We decided to include this patient in our cohort as the effect of 11q cannot be ruled out and it is essential to be aware that 11q trisomy patients may be prone to inborn errors of immunity as well. More research in this specific patient group is needed.

Also, although a study design with a standardized protocol was followed, we were not able to avoid missing values with regard to clinical data and laboratory test results. Still, we think that study results are applicable to other 11q patients as specific clinical symptoms and immunological laboratory results were more frequently observed than expected in the general population.

Lastly, we were not able to organize serological testing for CMV infections retrospectively due to ongoing IgRT or lack of stored plasma. Therefore, it is more difficult to draw definite conclusions on the results of the OX40-based T lymphocyte function test. We consequently recommend treating physicians to test EBV, CMV seroconversion before T lymphocyte function is tested or before IgRT is started to enable more clear conclusions in the near future.

Overall, we conclude that patients with partial 11q deletion or 11q duplication regardless of involvement of genes as *ETS1* or *FLI1* have a risk of inborn errors of immunity, consisting of both quantitative and qualitative defects. We therefore recommend regular immunological screening by testing IgG, IgA, and IgM, response to immunization, and B and T lymphocyte counts in all patients with 11q disorders. It is important to realize that lymphocyte dysfunction does not always correlate with B and T lymphocyte count. When newborn screening by T lymphocyte receptor excision circle (TREC) is performed, T lymphocytopenia should promptly lead to further investigation involving array analysis, copy number variation, or comparative genomic hybridization. [44, 45]. Immunological abnormalities may develop over time and need repetitive testing. In the case of a humoral immunodeficiency, prophylactic antibiotics and/or IgRT should be considered based on number and severity of infectious complications and potential end-organ damage. In the case of chronic fungal infections, especially affecting skin or nails, local treatment or systemic prophylaxis is indicated. Physicians should be aware that fungal infections and prolonged viral infections may still occur while on IgRT therapy.

As many questions remain in this intriguing immunological field, future research should focus on pathophysiological understanding of this combined immunodeficiency in patients with 11q disorders which can be seen as a model to identify modifiers of immunological function in various disorders. Focus should be on other candidate genes that can influence the immunological dysfunctions seen in patients with 11q disorders.

## Supplementary Information

Below is the link to the electronic supplementary material.

Supplementary file1 (DOCX 22 KB)

## Data Availability

Data is derived from electronic patient file and research tests with consent of patients or caregivers.
