# Supplementary material for: Patients with Chromosome 11q Deletions Are Characterized by Inborn Errors of Immunity Involving both B and T Lymphocytes
Source: J Clin Immunol. 2022 Jun 28;42(7):1521–34. doi: 10.1007/s10875-022-01303-8 (PMC9674766; doi:10.1007/s10875-022-01303-8)
Supplement: Supplementary file 1 — Supplementary file1 (DOCX 22 KB) [file 10875_2022_1303_MOESM1_ESM.docx]

**Supplementary Table 1** Description of the cytogenetic alterations

| Patient  number | Diagnostic test | Chromosome | Basepares | MB | Probes |
| --- | --- | --- | --- | --- | --- |
| 1 | Array (Illumina Cyto-12 300x, Nexus 7.0) (GRCh18) | 11q23.3q25 | (119,956,891 -134,452,384) x1 |  |  |
| 2 | Karyotyping | 46,XX, del(11Xq23.3q25) |  | 15.5 |  |
| 3 | Array; (GRCh37) | 11q24.1q25  12q24.21q24.33 | (122,893,213 -134,450,518) x1  (114,633,820 -133,779,217) x3 | 12  19,2 | 755  1167 |
|  | Additional GTG FISH | 46,XY,der(11)t(11;12)(q24.1;q24.21) mat |  |  |  |
| 4 | SNP-array (CytoScan HD Array Affymetrix) (GRCh37) | 11q24.1q25 | (123,307,968 -134,938,470) x1 | 11.6 | 13938 |
| 5 | Array; (GRCh37) | 11q24.2q25 | (126728342 -134938470) x1 |  |  |
| 6 | Whole genome Array,  (Illumina HCS850k, Nexus); (GRCh19) | 11q24.2q25 | (124,552,720 -134,934,063) x1 |  |  |
| 7 | Micro-array, (Agilant 180K); (GRCh37)  Micro-array, (BlueGnome Cytochip v1,1, Bac-Arr CGH); (GRCh37) | 5q35.1q35.3  (RP11-20O22 🡪 RP11-451H23) x3  11q24.2q25  (RP11-168K9🡪RP11-469N6) x1 | (171063816-180099598) x3  (126456874-126971201) x1 | 10  9 | 10  10 |
| 8 | SNP-array (CytoScan HD Array Affymetrix); (GRCh37) | 11q24.2q25 | (126,908,896 -134,938,470) x1 | 8 | 9498 |
| 9 | Whole Genome Array  (Illumina Cyto-12 300k, Nexus 7.0, 1Mb CNV analysis array); (GRCh18) | 11q24.3q25 | (127,727,172 -134,452,384) x1 |  |  |
| 10 | Karyotyping <1985 | 46,XX,t(6p;11q) deletion 11q25 |  |  |  |
| 11 | Targeted array (Illumina HCS850k, Nexus); (GRCh19) | 11q14.1q22.1 | (79,729,111) -(98,148,532) x1 |  |  |
|  | Additional FISH | 46,XY,del(11)(q14-21q21-22)  (RP11-13L14) x1 |  |  |  |
| 12 | SNP-array (Cytoscan HD Affymetrix, Thermo Fisher) (GRCh 37) | 11q14.1q23.3 | (84,558,527 -117,346,256) x1 | 32 | 34144 |
| 13 | Karyotyping | 46,XX,+der(11).ish dup(11)(q21q23.3) x3 |  |  |  |
|  | FISH with DNA probe WCP11 (paint 11) 2005 | Dup(11)(q21-q23.3)  Probes 3.16 (11q21), NCAM (11q23.2) and 4.13 (11q23.3) x2 |  |  |  |
| 14 | Karyotyping | 47,XX,+der(22)t(11;22)(q23;q11) x3, mat |  |  |  |
